# Integrating teaching into routine outpatient care: The design and evaluation of an ambulatory training concept (HeiSA)

**DOI:** 10.3205/zma001158

**Published:** 2018-02-15

**Authors:** Jan Hundertmark, Sandra Karina Apondo, Jobst-Hendrik Schultz

**Affiliations:** 1 Clinic for General Internal Medicine and Psychosomatics, Heidelberg, Germany; 2 Clinic for General Psychiatry, Heidelberg, Germany

**Keywords:** teaching, ambulatory care, curriculum development, anamnesis, examination skills

## Abstract

**Background: **Direct patient contact is crucial in learning important interactional and examination skills. However, medical students have limited opportunity to self-responsibly practise these skills in authentic clinical settings and typically receive insufficient feedback on their performance. We developed a novel single-session ambulatory teaching concept (Heidelberg Student Ambulatory training, “HeiSA”) to prepare students more adequately for clinical-practical responsibilities.

**Methods: **To identify challenges and target group needs, we reviewed current literature and consulted an expert group of faculty lecturers and training researchers. The resulting course concept was put into practice at the University Hospital’s general-internistic outpatient department and evaluated in a pilot phase (winter term 2010, ten participants) and a main project phase (summer and winter terms 2011, 14 and 21 participants, respectively). Third and fourth-year students autonomously take a new patient’s medical history and conduct a complete physical examination in one hour under supervision, followed by extensive preceptor feedback. To assess learning achievements, participants and a control group self-rated their communication and examination skills before and (participants only) after the session on six-point Likert scales (1=completely able, 6=completely unable). The preceptor also evaluated the participants’ performance. Finally, all stakeholders re-evaluated the course concept.

**Results: **HeiSA is a feasible training concept and accepted by staff members and students. It provides opportunities to practise clinical skills in a relevant, authentic learning environment with extensive feedback. Participants report improved anamnesis (0.27±0.51,* p*=.003) and physical examination (0.25±0.41, *p*=.008) skills. The preceptor evaluated students’ performance to be generally high, with ratings ranging from 1.40±0.55 (item: the student does not interrupt the patient) to 2.51±0.89 (item: psychosocial anamnesis).

**Conclusions:** HeiSA is a viable course concept for teaching anamnesis and physical examination skills. It integrates student teaching into routine care and can potentially be adapted to other outpatient departments.

## Zusammenfassung

**Hintergrund: **Beim Erlernen wichtiger ärztlicher Interaktions- und Untersuchungsfertigkeiten spielt direkter Patientenkontakt eine wesentliche Rolle. Dennoch haben Studierende der Humanmedizin typischerweise nur wenig Gelegenheit, diese Fertigkeiten im klinischen Umfeld eigenverantwortlich zu üben oder erhalten wenig Rückmeldung zu ihrer Leistung. Ein neuartiges Trainingskonzept für den Ambulanzunterricht, die „Heidelberger Studentische Ausbildungsambulanz“ (HeiSA), bereitet Studierende zielgerichtet auf praktische klinische Tätigkeiten vor.

**Methode:** Zur Bedarfsanalyse sowie zur Identifikation potenzieller Herausforderungen und Chancen wurden eine Literaturrecherche durchgeführt und in Kooperation mit einer Expertengruppe aus Hochschuldozenten und Lehrforschern ein Lehrkonzept für den Ambulanzunterricht entwickelt. Dieses wurde in einer Pilotphase (Wintersemester 2010, 10 Teilnehmer) sowie einer Hauptphase (Sommer- und Wintersemester 2011, jeweils 14 bzw. 21 Teilnehmer) umgesetzt. Bei HeiSA erheben die teilnehmenden Studierenden des sechsten und siebten Fachsemesters die Anamnese eines erstvorstelligen Patienten und führen anschließend eine komplette körperliche Untersuchung durch. In dem vorgegebenen Zeitrahmen von einer Stunde agieren die Studierenden eigenständig unter Supervision des Dozenten, der anschließend ausführliches Feedback gibt. Die Teilnehmer sowie der Dozent bewerteten Kommunikations- und Untersuchungsfertigkeiten vor und nach der HeiSA-Sitzung auf sechsstufigen Likertskalen (1=„Stimme voll zu“, 6=„Stimme gar nicht zu“); eine Kontrollgruppe wurde erhoben. Abschließend wurde HeiSA von allen Beteiligten re-evaluiert.

**Ergebnisse: **HeiSA ist ein praktikables und allseits gut angenommenes Lehrkonzept, das die Übung klinisch-praktischer Fertigkeiten in einer authentischen, relevanten Lernumgebung inklusive ausführlichem Dozentenfeedback ermöglicht. Die Teilnehmer berichten verbesserte Kompetenzen in Anamnese- (0.27±0.51, *p*=.003) und körperlichen Untersuchungstechniken (0.25±0.41, *p*=.008). Der Dozent beurteilte ihre Leistung als durchgängig gut, mit Bewertungen von 1.40±0.55 bis (Item: „Lässt den Patienten aussprechen“) bis 2.51±0.89 (Item: „Vollständige psychosoziale Anamnese“).

**Schlussfolgerung: **HeiSA ist ein sinnvolles Lehrkonzept zur Vermittlung klinischer Kommunikations- und Untersuchungsfertigkeiten. Es integriert universitäre Lehre in die Routineversorgung und kann potenziell in anderen Klinikambulanzen eingesetzt werden.

## Background

Medical training is aimed at enabling future physicians to autonomously and responsibly carry on their profession. However, medical students and graduates generally do not feel adequately prepared for their work in terms of communicational and clinical-practical skills [1]. Some studies show that students are insufficiently taught to conduct a clinical examination in a systematic, structured, and hypotheses-based manner [2], [3], [4], [5]. Direct patient encounters are crucial in the development of these abilities, especially if they are perceived as real and relevant. Diemers et al. [6], [7] further found them to motivate students to study, help them understand the impact of illness on patients’ lives, and enhance professional socialisation as well as integration of theory and practice (also [8]). Similarly, Dornan et al. [9] showed in a systematic review that early patient contact strengthens learning in a range of areas and stimulates students to develop confidence, self-reflection, and a professional identity, including specialisation choices. Patient contact in medical education typically takes place as bedside teaching, which is commonly used to practise examination techniques, but also allows for training communication and interaction skills [10], [11]. However, students generally see the potential of this teaching setting as not exhausted, due to limitations in autonomy and available time with the patient, little responsibility, and insufficient supervision and feedback [12].

Ambulatory settings, in contrast, are much less frequently made use of for student teaching, even though researchers have been demanding their increased use for decades [13], [14], [15], [16], [17]: Patients present with a large variety of symptoms that are more representative of general medicine practice and require diverse diagnostic measures and interventions [18], [19]. Teachers can choose from a plenitude of potential learning objectives that typically require students to develop and apply both communication and examination skills [20]. A feature unique to ambulatory settings is the possibility for students to take responsibility for initial clinical contacts with patients that have not yet been diagnosed – a relevant, authentic, and therefore motivating and rewarding experience [21]. Although studies comparing student performance after hospital-based vs. ambulatory clerkships found no significant differences [21], [22], students do seek more “hands-on” contact to patients and enjoy initial examinations without diagnostic appliances [23]. They generally appreciate self-reliant interaction with outpatients and experience a stronger sense of responsibility and identification with the physician role, in comparison to inpatient wards [21].

Nevertheless, students easily feel overchallenged and apprehensive in ambulatory settings [24] and demonstrate higher stress levels than in in-hospital settings [25]. High-quality instruction and supervision seem to be crucial: According to students, successful learning depends on clarity about procedures and expectations, the preceptor’s encouragement, enthusiasm, constructive feedback, and his ability to provide a positive role model and comprehensively explain clinical decisions [24], [26], [27], [28]. As to the concrete implementation of ambulatory trainings, researchers report a number of pitfalls. The diversity of encountered symptoms, conditions, and procedures complicates the standardisation of learning goals [20]. Structure in teaching is often insufficient, as case discussions with attending physicians do not take place or are short, with little actual teaching and almost no feedback. Common reasons for these shortcomings are time pressure and large numbers of patients [18], [29], which in combination with teaching assignments leads to stress and overload of the teaching physicians [30] or increased operating costs [31], [32]. In sum, Irby [20] concluded in a comprehensive literature review that teaching and learning in this setting was suboptimal and characterised by “variability, unpredictability, immediacy, and lack of continuity”. To promote effective and gratifying teaching in outpatient settings, McGee and Irby [23] suggest several practice points: clear instructions about the task and expectations, priming for important aspects of the consultation (e.g. emphasis on differential diagnosis), asking questions and showing interest in the student’ thoughts, setting a focus on one teaching point, modelling and vocalising during the teacher’s own patient interactions, and providing feedback to the learner. Taken together, successful teaching in ambulatory settings requires a balance of conceptual scaffolding by the teacher, self-reliant practice by students, and meaningful reflection and dialogue between them.

In the US, pre-clinical training in ambulatory setting has been taking place in student-run outreach clinics. Within the last two decades, in the context of recent crises in cost, quality of care, as well as high uninsurance rates [33], [34], [35], at least 62% of US medical schools have established at least one of these clinics [36]. They typically operate once a week, offering acute treatment, management of chronic health problems, or physical check-ups for dispriviledged patients under supervision and assistance of licensed healthcare professionals [35], [37]. Students generally enjoy their (voluntary) participation in these clinics and report increases in knowledge, skills, and motivation; however, objective and long-term effectiveness measures have not been sufficiently assessed [37], [38], [39], [40]. Even though student-run clinics offer various potential learning opportunities beyond the formal curriculum, explicit teaching is often sparse or unsystematic [38], [39], and many of the above shortcomings still seem to apply. In Germany, due to large differences in public health policy and lower uninsurance rates, the necessity and feasibility of student-run clinics is much lower; therefore, only one similar centre has been established so far [41].

To harness the potential benefits for practical, competency-based education described in literature within our framework conditions [42], [43], we designed the Heidelberg Student Ambulatory training (Heidelberger Studentische Ausbildungsambulanz, “HeiSA”), a single-session training concept that can easily be implemented into routine care procedures. Participating students take on the role of an outpatient department physician and autonomously welcome a new patient in one single encounter, take his medical history, and conduct a complete physical examination. The responsible attending physician is present to supervise and provide the student with detailed feedback. This study aims to outline HeiSA’s development procedure, present the resulting training concept, report first experiences, and assess its training effect. We hypothesised that HeiSA has positive effects on participants’ anamnesis and physical examination skills.

## Method

### Study design

HeiSA’s conceptual development started in 2010 and roughly followed the six-step Kern [44] cycle of medical curriculum development: First, we reviewed current literature as summarised above. We then consulted an expert group of faculty lecturers and training researchers to identify given shortcomings and assess both general and target group needs. Afterwards, the expert group defined learning goals and objectives, again with reference to research literature, and developed according educational strategies. In this process, it focussed on complementary, practice-oriented teaching methods different from those already employed in the pre-existing Heidelberg medical curriculum with its well-structured lead-symptom oriented lectures and patient contact mainly taking place in university hospitals wards or with simulation patients [42], [43]. Furthermore, by integrating student training into running routine care activities, the expert group specifically aimed to avoid the logistical and structural challenges common to ambulatory trainings. An implementation plan including a concrete schedule resulted and was put into practice in a pilot phase with ten participants in the winter term 2010/2011. As the last step of the curriculum development cycle [44], the expert group revisited all steps and consulted relevant stakeholders to evaluate the project’s organisational feasibility and assess the preceptor’s and students’ satisfaction. These consultations were not systematically recorded; however, they produced valuable suggestions and solution approaches that led to adaptations of logistical procedures, teaching methods, and learning goals. The improved concept was realised and re-evaluated in a main project phase in the two subsequent summer and winter terms of 2011 to 2012.

We assessed students’ skills and performance using quantitative self-report and preceptor ratings at HeiSA sessions. The preceptor was a training assistant and a third-year registrar in general-internal medicine. As attending physician, he was responsible for patients’ treatment and ensured HeiSA’s consistency. Moreover, to examine self-selection effects, we gathered data from a control group comprised of same-year students who did not participate in HeiSA: All students who were present on one selected day during the first week of their psychosomatics module were asked to participate in the study and fill out self-rating questionnaires.

#### Learning goals

HeiSA attends to the following educational needs in the areas of communication, examination, and professional role development.

- Establishing a positive initial contact with a patient.
- Reviewing a case file and understanding its gist.
- Properly managing time in anamnesis and physical examination.
- Autonomously conducting an anamnesis and physical examination.
- Adequately using interviewing techniques.
- Developing own ideas concerning diagnostics and therapy.
- Reflecting on own performance, abilities, and professional role.
- Accepting feedback and criticism.

#### Educational strategies

The following setting and educational strategies were chosen to optimally support students to reach the above learning goals.

- Students autonomously prepare and take a patient’s history.
- The preceptor watches the student’s anamnestic interview through a one-way mirror.
- Students perform a physical examination under supervision.
- Extensive debriefing and feedback directly after the examination.

#### Instruments

We used quantitative questionnaire to assess students’ abilities. The students’ version had pre- and post-session-ratings, in which participants self-rated their perceived current skills in different aspects of physical examination as well as communication and interaction (4 and 6 items, respectively; see table 1 (Tab. 1) for a complete list) on six-level Likert scales ranging from 1=*completely able* to 6=*complete unable*. The preceptor rated students’ skills after their anamnesis and examination, respectively (altogether 20 items in an extended but comparable version of the students’ questionnaire). The control group questionnaire was identical to the participants’ pre-session version. Finally, we calculated scales for self-reported (anamnesis, physical examination) and preceptor-rated skills (anamnesis, patient interaction, physical examination).

#### Participants

HeiSA aims at students in their sixth and seventh semester who currently take the internal medicine block lessons. Participation was voluntary but linked to participation in this study during its course. Excluding the pilot phase, 35 students (mean age 22.6, 18 women) participated, 14 in the summer term and 21 in the winter term. The control group comprised 29 students, 15 in the summer term and 14 in the winter term.

#### Inclusion criteria and patient allocation

HeiSA is open for up to 30 voluntary participants. Course registration is organised by participant lists and follows a “first come first served” principle.

The allocation of patients is organised by the internistic medicine outpatient department coordination office. Its staff extensively explained the project to patients, obtained a first consent and suggested an appointment. Patients were not preselected and age, gender, or the patient’s suspected diagnosis were not taken into account; therefore the single inclusion criterion (next to voluntary participation) for patients is first contact to the department.

#### Procedure of a HeiSA-session

HeiSA follows a standardised session procedure, as described in this paragraph and summarised in table 2 (Tab. 2). Up to three subsequent sessions take place on one day of the week. The examination room and materials are prepared in advance. The coordinating office rechecks the patient’s consent and hands the case file to the preceptor. The student acquaints himself with the examination room, reviews the case file, and completes the first version of the self-assessment questionnaire. The preceptor and student briefly discuss the case file (ca. 2 minutes) and identify the cardinal symptom as well as the referring physician’s suspected diagnosis.

On his arrival, the patient is welcomed by both student and preceptor, who again explains the procedure, assures the patient’s consent, and then leaves the room. The student autonomously takes the patient’s medical history. The preceptor watches through a one-way mirror, takes notes of the student’s performance, and gathers diagnostic information for his own records. He re-enters the examination room when the student finishes the anamnesis, usually after a maximum of 20 minutes, and complements the anamnesis with own questions if necessary. As patients are undiagnosed, thus unselected, and their cases potentially challenging, students may ask the preceptor to return earlier to support them at any time. However, the general rule is that he only interferes on his own initiative when students pass a time limit of 45 minutes, in the case of grave misunderstandings, or when imminent conflicts appear, which the student apparently cannot solve.

Upon completion of the anamnesis, the student again takes charge and bridges to the physical examination, which he conducts autonomously in terms of extent, accuracy, and technique. He is allowed, however, to ask the preceptor for suggestions or explanations at any time if necessary. Again, the preceptor stays in the background and only intervenes in the case of grave mistakes or incomplete examinations. The student is free to comment on the examination procedure and mention normal results; however, he is obliged to voice pathological or suspicious findings to be noted down. He furthermore has to abide by the one-hour time frame common in the department and end the examination in agreement with the preceptor, who then communicates further measures, such as taking a blood sample, ECG, or sonography, to the patient and the coordination office.

After dismissing the patient, the preceptor and student discuss the case and the student’s behaviour. The student recapitulates the case, starting with personal reflections on his experiences and impressions and finishing with own ideas concerning further diagnostic and therapeutic measures. He then receives an in-depth feedback about his performance. This may bridge to a discussion about the patient-doctor-relationship, conversation techniques, or medical details about syndromes, results, or examination methods. Finally, both preceptor and student complete an assessment questionnaire and close the session.

## Results

### Feasibility and acceptance

The expert group’s consultations of relevant stakeholders indicate that the implementation of HeiSA, an ambulatory training in the internistic medicine outpatient department of Heidelberg university hospital, was successful. Teachers and hospital staff were satisfied and had positive attitudes towards the programme. Both sufficient patient care and supervision could be ensured, but HeiSA required a certain degree of support by the department staff: Its scientific lead was taken by the department’s medical director, who authorised the restructuring of facility routines, and a senior physician, who supervised the preceptor and the project coordinator. The patient throughput on days with HeiSA sessions was slightly lowered.

However, after the project’s pilot phase, some logistic and organisational challenges were reported to the expert group and subsequently amended: The date for HeiSA sessions was moved to the morning, so that further examinations could take place on the same day. Clear arrangements with the department’s coordination office proved important to arrange patient assignment, minimise disturbances in routine operations, and thus ensure continuity in patient treatment after HeiSA. Another early challenge was timing: After initial negative experiences, students were instructed to arrive earlier to review the case file and prepare for a cardinal symptom-oriented approach. Moreover, in the pilot phase the preceptor was sometimes forced to repeat large parts of history taking or examination. Later on, due to better coordination with the student, he could save time by confining himself specifically to complementing questions and examinations. The clear one-hour time frame then proved sufficient for both teaching and patient treatment.

The preceptor reported that participants express high appreciation of the entrusted responsibility, the opportunity for practice and reflection, and the extensive feedback they receive. Students generally performed well during history taking, but as patients were previously undiagnosed, occasionally they indeed felt unable to complete their task on their own. First impressions are that this did not impair learning, but, to the contrary, added to the relevance and authenticity of the setting and offered the preceptor the opportunity to pose complementary questions, serve as a role model, and discuss the according situation in the subsequent feedback. In physical examinations, students were mostly well prepared and sufficiently skilled. Therefore, preceptor interventions were usually limited to suggestions or checking questions, with the exception of auscultations of heart or lungs, which he frequently had to repeat.

#### Self-assessment of student abilities

To assess whether HeiSA-participants differ from non-participating students, we performed Mann-Whitney tests on all dependent variables, revealing no differences in self-rated history taking and examination skills before HeiSA (all *U’s*≥370.5, n.s., *n_1_*=35, *n_2_*=29).

Table 3 (Tab. 3) provides a summary of students’ ratings and results of pre-post comparisons using Wilcoxon signed-rank tests. The tests reveal that HeiSA-participants report significantly improved abilities in medical history taking (improvement 0.25 on anamnesis scale, all test statistics shown in Table 3 (Tab. 3)), in particular to identify patients’ psychosocial stressors (0.40), deal with their psychosocial problems (0.49), and establish a viable doctor-patient relationship (0.66). As to physical examination skills, mean ratings significantly improve as well (0.27), in particular the ability to conduct a complete physical examination in a structured manner (0.38).

#### Preceptor assessment of student abilities

Table 4 (Tab. 4) shows the supervising preceptor’s rating on 20 variables. He rated students’ performance as generally high, with mean ratings ranging from 1.40±0.55 (item: the student does not interrupt the patient) to 2.51±0.89 (item: complete conduction of a psychosocial anamnesis). Mean scale scores are 1.86±0.48 for anamnesis, 1.71±0.47 for patient interaction and 1.77±0.74 for physical examination skills.

## Discussion

Researchers typically report various shortcomings of student teaching in ambulatory settings, such as lack of structure or continuity, overload of teachers, or insufficient supervision and reflection. We aimed to develop an ambulatory training concept that complements existing teaching methods at Heidelberg University and addresses most of the above shortcomings. First experiences show that HeiSA is one viable approach that offers a clear structure, a relevant and motivating setting, and sufficient time for supervision and feedback by integrating student teaching into routine care. It furthermore provides an example of another successful application of the Kern cycle in medical curriculum development.

The study’s major limitations are the assessments of training effects as well as students’ experiences. Students’ mean self-rated abilities in medical history taking and physical examination improved after participating in HeiSA, indicating a positive learning outcome. However, self-assessment does not necessarily reflect learning and despite its common usage, evidence for correlations of self-ratings and actual behaviour in clinical practice is still sparse [45], [46]. Furthermore, students’ rating may be influenced by higher self-awareness or self-criticism after the training, a phenomenon well known in medical education literature [47], [48]. Now that HeiSA as a feasible ambulatory training concept is established, future research should aim to assess positive effects more validly, for instance in OSCEs or other clinical performance measures.

Moreover, some learning outcomes are difficult to assess using quantitative measurements. For instance, the responsibility in authentic contacts with undiagnosed patients and the subsequent teacher feedback potentially stimulate students’ self-awareness and professional identity development. Questionnaires or clinical examinations are ill-suited to elicit these aspects of learning, which nevertheless are crucial for successful socialisation into the physician role. Again, further systematic research, ideally by means of qualitative interviews, is necessary to determine HeiSA’s concrete effects on self-reflection and its potential for professional role formation. This research should furthermore attempt to corroborate students’ appreciation of the HeiSA programme and explore to what extent it complements the pre-existing medical curriculum.

Other possible methodological limitations to this study are ceiling effects due to the preceptor’s high ratings and sample effects. Neither the HeiSA participant group nor the control groups were representative, and even though we found no significant differences between groups, the sample’s representativity may be limited by self-selection: As participation in HeiSA and this study were linked and followed a first come first served principle, participants might have been especially motivated and involved. However, preceptor assessment was only a secondary measure and direct comparability not an objective in this study.

## Conclusions

In the current study, we presented the Heidelberg Student Ambulatory training (HeiSA) as a viable course concept integrating teaching into routine patient care. HeiSA offers opportunity to practise anamnesis and physical examination skills in an authentic, relevant and responsible setting. It provides sufficient student supervision as well as continuity in patient treatment. First project evaluations show positive results and demonstrate HeiSA’s feasibility as well as high general acceptance by students and department staff. HeiSA is a successful teaching concept that can potentially be applied in and adapted to other outpatient departments.

## Declaration

### Acknowledgements

We acknowledge financial support by Deutsche Forschungsgemeinschaft and Ruprecht-Karls-Universität Heidelberg within the funding programme Open Access Publishing.

#### Ethics

All research has been conducted in accordance with the Declaration of Helsinki and has been approved by the ethics committee of the Medical Faculty of Heidelberg University (reference number S-262/2014). All participants were informed orally and in writing about the proceedings of the study and gave their informed consent. No individual participant or patient data are reported in this study.

#### Authors’ contributions

SKA developed the educational project, recruited participants, and overviewed all project stages. JHS supervised the project development and participated in the expert group. SKA and JH searched literature and conducted data analyses. JH prepared the manuscript. All authors read and approved the final manuscript.

## Competing interests

The authors declare that they have no competing interests. 

## Figures and Tables

**Table 1:**
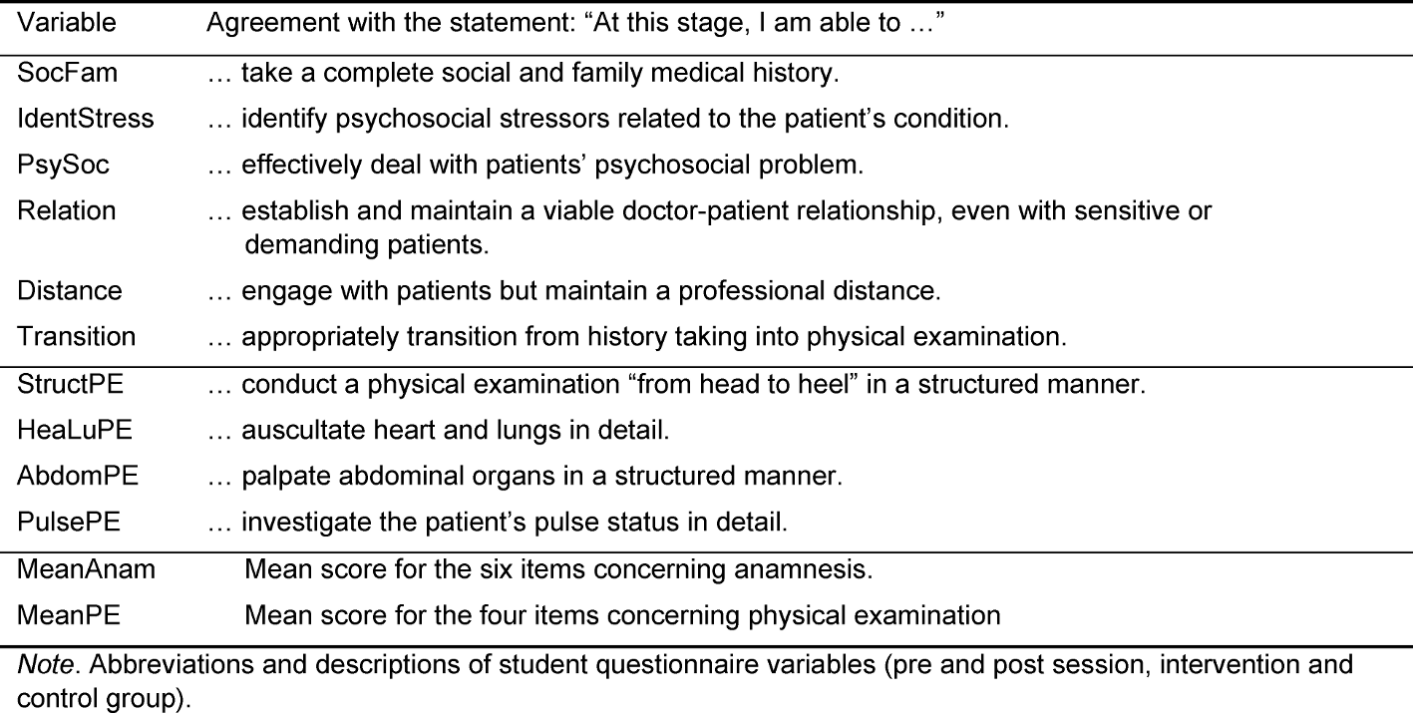
Participants’ self-rating questionnaire: variables and item descriptions

**Table 2:**
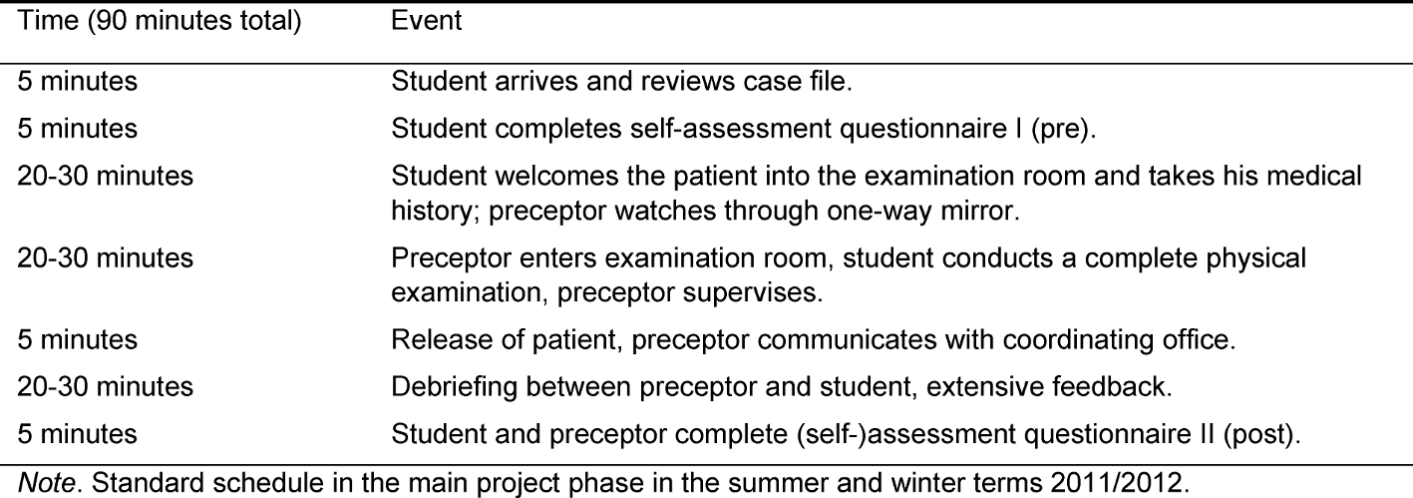
Schedule of a HeiSA session

**Table 3:**
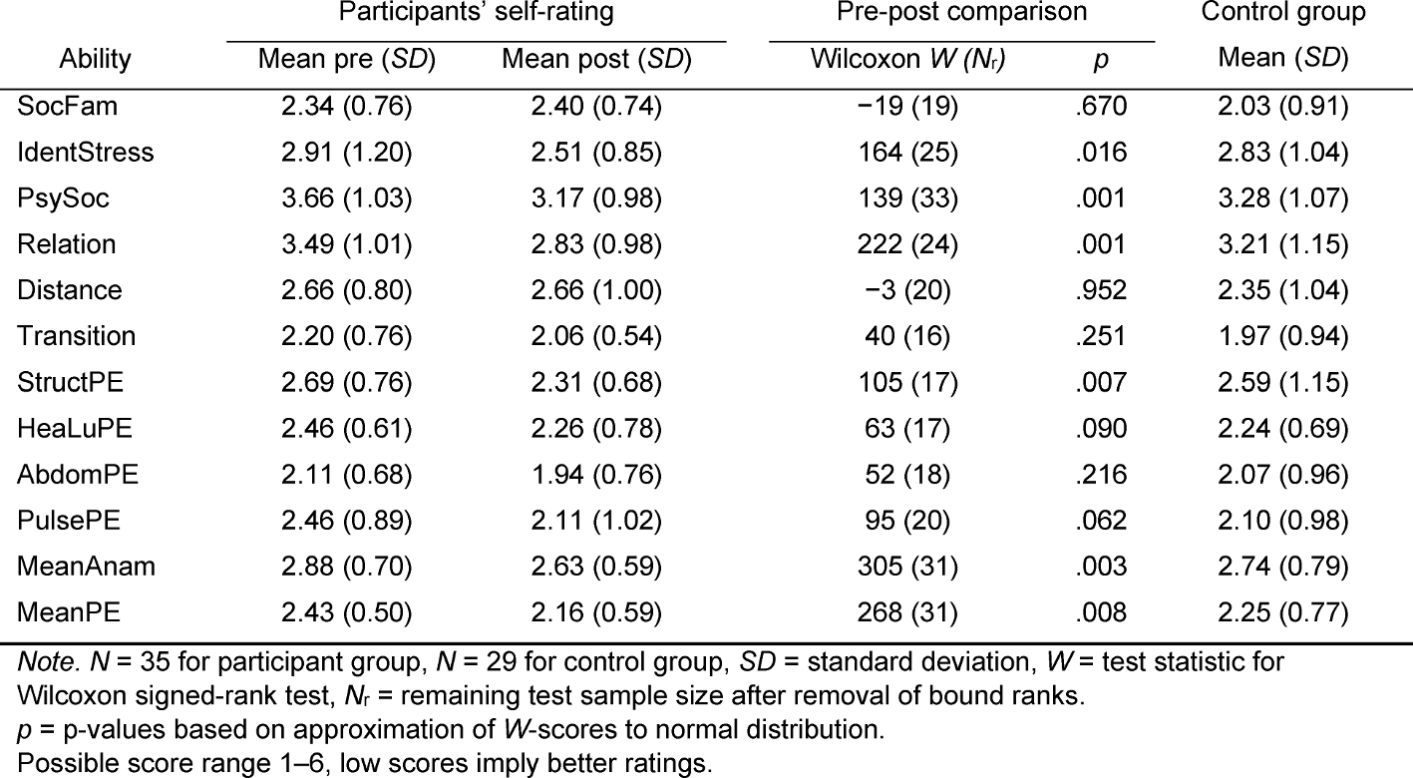
Participants’ self-assessment results and pre-post competency comparisons

**Table 4:**
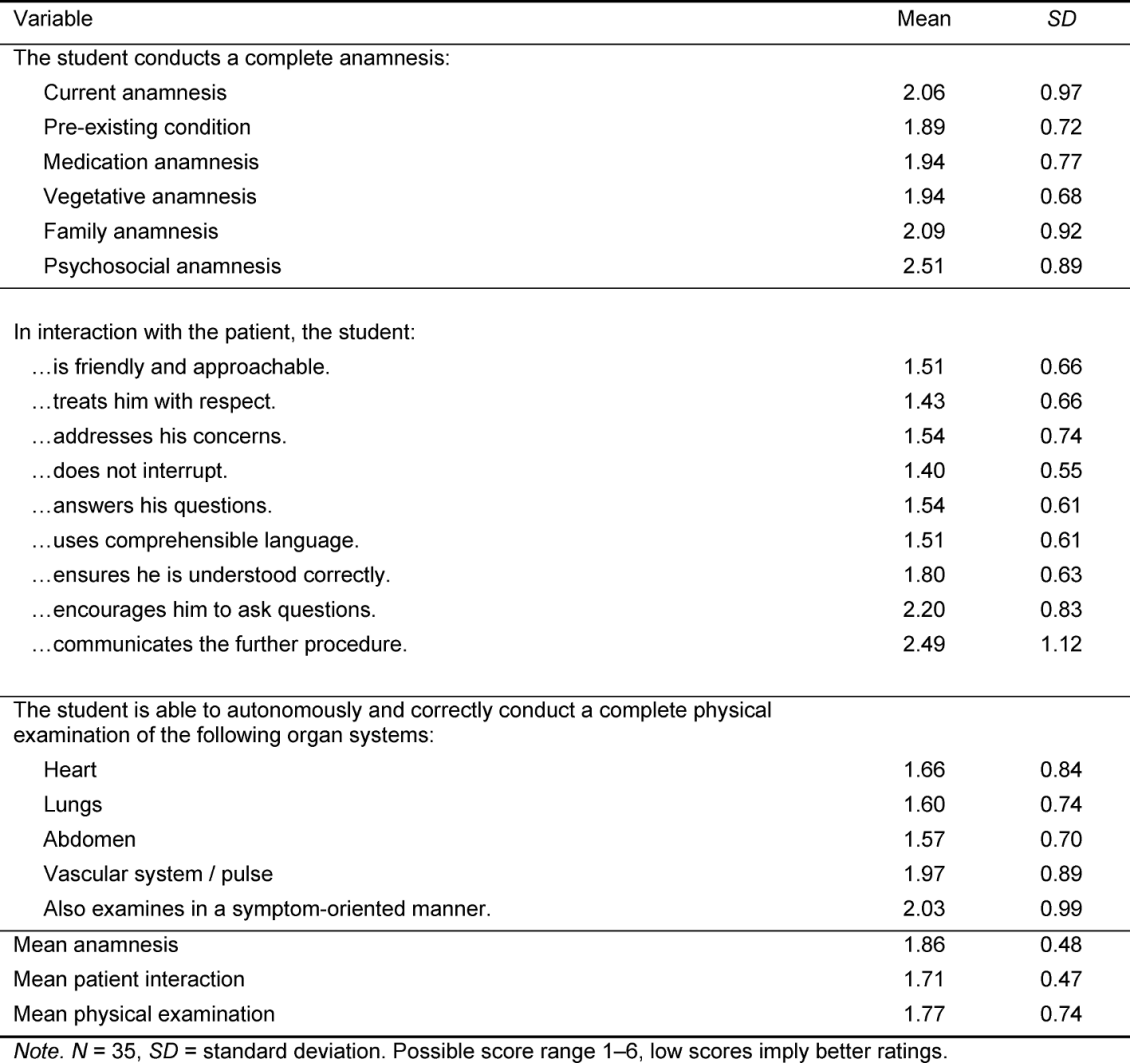
Preceptor assessment of participants’ competencies
